# Stability analysis of dam grouting reinforcement based on FLAC3D

**DOI:** 10.1371/journal.pone.0342938

**Published:** 2026-02-27

**Authors:** Pengfei Qin

**Affiliations:** Engineering Department of Huanghe University of Science and Technology, Zhengzhou, China; SASTRA Deemed University, INDIA

## Abstract

Ill reservoirs have significant safety hazards, it is urgent to grout and strengthen the dangerous reservoir dams. In order to effectively evaluate the stability of the dam after grouting reinforcement, this paper carried out numerical simulation analysis work. Firstly, the action mechanism of penetration grouting, compression grouting and splitting grouting is described, and it is pointed out that these modes of action are intermixed and interconverted, and they may exist simultaneously in the same grouting process. Next, based on the FLAC3D finite difference program, the analysis and calculation of the stability of seepage control reinforcement of a dangerous reservoir in Guangxi Province were carried out. The calculation results show that, a complete grout curtain zone is formed inside the dam body after grouting reinforcement treatment, the dam stress is adjusted twice, the area of plastic zone decreased 23%, and the stability of the dam body is significantly enhanced. This article has certain guiding significance for the analysis and evaluation of safety and stability of similar projects.

## 1. Introduction

Dams play an important role in irrigation, power generation, flood control and other fields. However, many dams have experienced leakage due to long-term overload operation [1,2]. Reservoir leakage will bring significant losses to the economic benefits of the project and even affect the safety and stability of the dam. The water level difference between the upstream and downstream of the dam is large, and the head difference between the inside and outside of the dam will produce high permeability in the foundation rock and soil, thus causing seepage damage phenomena such as submerged erosion, quicksand, or piping. Statistics on dam accident investigations at home and abroad show that engineering accidents caused by leakage damage account for more than 40% [3,4].

Grouting reinforcement of dam foundation and dam body is an important technical means to solve seepage damage. After the slurry is injected into the rock and soil layer, it can form a stone body with high strength, new structure and good stability, which can significantly improve the bearing capacity and anti-seepage performance of the foundation. ‌With the continuous expansion of the development scale of underground engineering construction and the construction of water conservancy projects, the grouting technology has been more and more widely used. The paper describes the common grouting action mode, and then based on FLAC3D finite difference program, the numerical analysis of the reinforcement effect of a water conservancy project in Guangxi Province is carried out. The paper thoroughly and comprehensively evaluates the stability of the dam by comparing the distribution of the maximum and minimum principal stresses, as well as the changes in horizontal and vertical displacements before and after grouting, demonstrating certain theoretical innovations.

## 2. Grouting mode of action

According to the mode of action of grout and foundation soil during grouting, the grouting technology can be divided into penetration grouting, compression grouting and splitting grouting.

### 2.1. Penetration grouting

**Fig 1:**
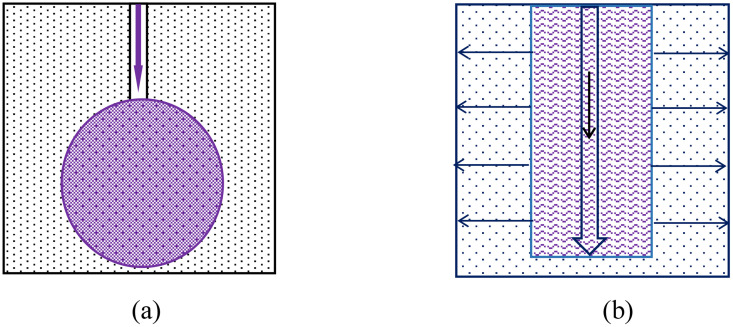
Spherical diffusion mode (a); Cylindrical diffusion mode (b).

Penetration grouting is to inject slurry into the pores of granular soil under a pressure that is not sufficient to destroy the formation structure for the purpose of seepage control and water plugging or formation reinforcement [5–7]. During penetration grouting,the slurry diffusion pattern is uniform, and the form of slurry diffusion depends on the shape of the grouting pipe flower hole. If end grouting is used, the slurry will diffuse in a spherical manner,if flower-tube segmented grouting is used, the slurry will diffuse in a cylindrical manner, as shown in Fig 1.

There are time-varying viscosity effects, tortuosity effects and percolation effects in the slurry penetration and diffusion process [8–10]. The time-varying viscosity effect refers to the continuous change of grout viscosity with the grouting time, the tortuous effect means that the diffusion path of grout in the pore channel changes tortuously, and the percolation effect means that granular materials continue to filter out and accumulate on the grouting channel. The diffusion radius of the slurry can be calculated using Maag’s formula.


$$R=\sqrt[{\text{3}}]{\frac{\text{3}f_{\text{0}}a_{\text{0}}kt}{\lambda n}}$$ (1)

$$R=\sqrt{\frac{\text{2}f_{\text{1}}kt}{\lambda n\text{ ln}\frac{R}{a_{\text{0}}}}}$$ (2)

Where *R* is slurry diffusion radius (cm), *k* is sand soil permeability coefficient (cm/s), *f*_0_ and *f*_1_ are grouting pressure head (cm), *a*_0_ is grouting hole radius (cm), *t* is grouting time (s), *λ* is viscosity ratio of slurry to water, *n* is porosity of sand soil.

### 2.2. Compaction grouting

Compaction grouting uses higher pressure to inject cement slurry or chemical slurry with a higher concentration into the formation. During compaction grouting, as the grout continues to be injected, grout bubbles are generated near the end of the grouting pipe. The initial diameter of the slurry bubble is small, and the slurry radiates and spreads in the formation along the radial direction with the grouting hole as the center. The grout replaces the soil in the grouting range, and there is a large plastic deformation zone near the grouting hole, while the soil in the area far away from the grout bubble undergoes elastic deformation. Under the action of compaction effect, the density of the soil layer increases significantly, and as the size of the slurry bubble increases, the ground slightly lifts [11,12].

**Fig 2:**
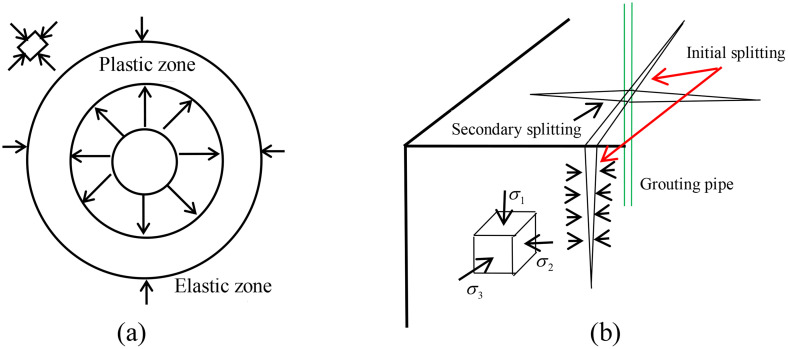
Schematic diagram of compaction grouting reaming (a); Schematic diagram of splitting grouting (b).

Compaction grouting is suitable for sandy soil foundations and clay foundations with good drainage conditions. If the drainage conditions in the clay foundation are poor, the pore water pressure is difficult to dissipate, and the grouting rate needs to be appropriately reduced during compaction grouting. After reinforcement by compaction grouting, the anti-seepage performance and load-bearing performance of the stratum are significantly improved. Therefore, compaction grouting can be widely used in projects such as regulating uneven settlement of buildings, controlling lateral deformation of tunnels, and preventing subgrade collapse(Fig 2a).

### 2.3. Splitting grouting

#### 2.3.1. Principle of reinforcement.

Splitting grouting is the most widely used foundation reinforcement method in dam foundation treatment [13,14]. The principle of splitting grouting is, applying continuously increasing grouting pressure in the weakly permeable foundation, when the grouting pressure reaches the initial splitting pressure of the soil layer, it will create splitting joints in the soil body, and the pressure exerted by the slurry on the splitting surface will then push the cracks to open and expand rapidly. Please see Fig 2(b). With the continuous increase of grouting pressure and the continuous injection of slurry, the slurry in the soil body forms longitudinal and horizontal mesh of slurry veins near the borehole, and the normal stress and stiffness of the soil are significantly increased by squeezing the soil through the formed veins and reinforcing it with the skeleton effect of the veins.

#### 2.3.2. Action process.

According to the different grouting pressures, the interaction mechanism between the splitting grout slurry and the soil body can be divided into three stages: the initial bubble compacting stage, the splitting flow stage and the passive earth pressure stage [15,16].

(1) Initial bubble compacting stage. At the beginning of grouting, the slurry pressed out from the grouting pipe will first fill the space between the outer wall of the grouting pipe and the hole wall of the grouting hole, and because the energy possessed by the slurry is not large enough to split the stratum, the slurry gathers near the hole of the grouting pipe and forms a spherical or columnar slurry bubble with the grouting pipe as the main body. With the continuous injection of slurry and the increasing of grouting pressure, the bubble gradually expands around and squeezes the surrounding soil, which belongs to the compression grouting stage.
(2) Splitting and flowing stage. When the soil around a certain depth in the foundation soil is first compressed and yielded to flow damage, the soil will be split by the grouting pressure and the slurry will split and flow in the stratum. The splitting surface occurs at the small main stress surface where the resistance is minimal, and at this time the slurry bubbles will be interconnected, and forming a longitudinal and horizontal mesh of slurry veins in the soil.
(3) Passive earth pressure stage. As the crack propagates in the formation and grout continues to be injected, the grouting pressure rises again. The soil stress field changes due to grouting, the large and small stresses in the formation transform into each other, and the horizontal principal stress transforms into a passive earth pressure state. As the amount of slurry injected continues to increase and accumulate, peak pressure occurs again in the soil layer. When the soil is in a soft state, the secondary peak pressure is not obvious. Under the action of horizontal stress, the formation produces staggered horizontal fractures.

It should be noted that the mode of action of infiltration, compacting or splitting between slurry and soil body does not exist in isolation, but is mixed and transformed with each other, and they may exist in the same grouting process at the same time. In low pressure grouting, the main mode of action is infiltration, while in high pressure, the mode of action is splitting and compacting. Where the splitting grouting is a process of first compression and then splitting, and the splitting grouting is accompanied by other grouting modes such as compression and infiltration.

## 3. FLAC3D calculation method

FLAC(Fast Lagrangian Analysis of Continua) is one of the excellent geotechnical numerical calculation software systems in the world, and FLAC3D is a three-dimensional finite difference program for rock and soil developed by Itasca Company of the United States on the basis of FLAC. FLAC3D is a numerical simulation technology based on “explicit Lagrangian” theory and “hybrid-discrete partitioning”, which is solved by hybrid discrete element method, dynamic relaxation method and explicit difference method [17,18]. FLAC3D has become an important tool for the numerical analysis of geotechnical problems because it does not form a stiffness matrix during the calculation process and has a fast computational speed with a small memory footprint.

**Fig 3:**
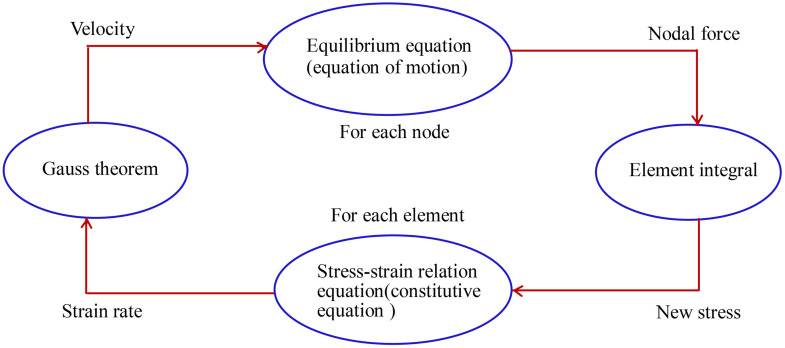
Schematic diagram of FLAC calculation cycle.

FLAC3D can simulate the deformation and damage behavior of media such as weathered rock and consolidated clay. FLAC3D divides the calculation area into four-node planar isoparametric units. The units follow corresponding linear or nonlinear constitutive relationships. If the unit stress causes the material to yield or produce plastic flow, the unit grid will deform or move accordingly. The main advantages of FLAC3D are as follows: (1) The “hybrid discrete method” is used to simulate plastic damage and plastic flow, which is more accurate and reasonable than the “discrete integration method” used by the finite element method; (2) The dynamic equations of motion are used to perform static and dynamic analysis and calculation of complex engineering problems, which is more efficient and scientific. FLAC3D supports the analysis of multi-physics coupling problems such as fluid mechanics and thermodynamics, and is currently widely used in fields such as geotechnical and underground engineering. The FLAC3D calculation principle is shown in Fig 3.

FLAC3D numerical simulation software is embedded with 12 mechanical constitutive models, including 1 excavation model, 3 elastic models and 8 plastic models, and its V2.1 version provides a good development environment, and users can also realize the secondary development of constitutive models in VC++ environment according to the calculation needs.

## 4. Engineering case analysis

Changtang Reservoir in Guangxi Province is a comprehensive Class II large-scale project mainly for ecological replenishment and spring drought water supply, and at the same time combined with power generation, with a total reservoir capacity of 238 million m^3^ and a total installed power plant capacity of 122 MW. The dam base cover layer of Changtang Reservoir is mainly composed of sand, pebbles and large boulders, with local overhead structure and strong permeability. No anti-seepage treatment was done during construction, and serious leakage occurred when water storage started, and multiple cracks appeared along the axis of the dam crest. For this reason, it was decided to use grouting technology for seepage control and reinforcement treatment. The grouting axis was arranged at the position of the dam axis after reconstruction, using plum-shaped holes with a distance of 3.6m and a depth of up to the design depth. The bank slope section uses double row staggered hole layout.

### 4.1. Establishment of three-dimensional numerical model

**Fig 4:**
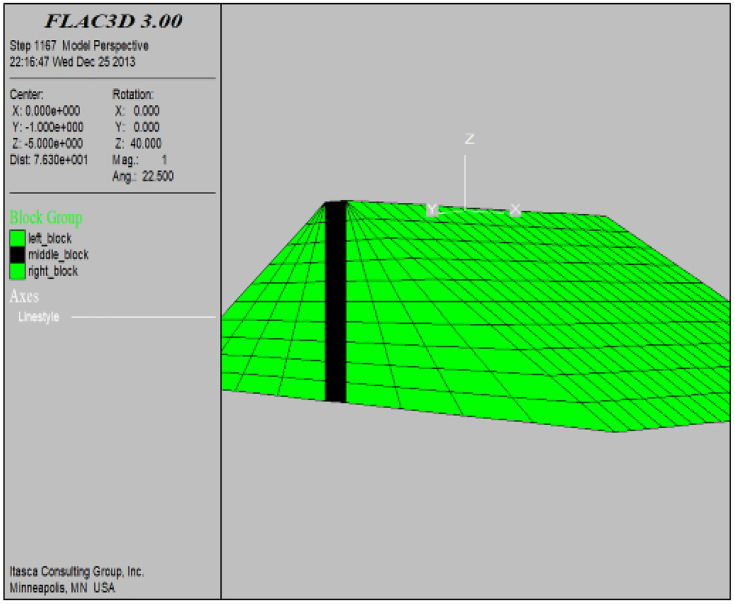
Calculation model.

In order to establish the numerical model of the dam, the right-handed coordinate system was selected to establish the three-dimensional coordinates and divide the finite-difference mesh elements. The coordinate origin is located in the middle of the dam crest axis, the height difference between the top and bottom of the dam is 21 meters, with a span of 226 meters for the dam body. The dam axis is taken as the X-axis, the horizontal direction perpendicular to the dam axis is the Y-axis, and the vertical surface direction is the Z-axis. In the boundary condition setting, the Z-direction displacement and velocity at the bottom of the dam body are fixed. The three-dimensional model has a total of 2,100 elements and 2,386 nodes, the computational domain is the entire dam body. Among them, the black part along the middle of the dam body axis is the grouting curtain zone and the rest is the non-grouting curtain zone, please see Fig 4.

### 4.2. Selection of constitutive model

In the numerical calculation of the reinforcement effect of anti-seepage grouting for dams, the stability of the dam is changed due to the injection of a certain amount of slurry into the dam body, and the mechanical parameters that best reflect the stability of the dam body, cohesion *c*, and internal friction angle *φ*, will be improved to varying degrees. In this numerical simulation, the Elastic Model is used for the grouting curtain zone, and the Mohr-Coulomb Model is used for the non-grouting curtain zone.

The yield criterion of the Mohr-Coulomb model is


$$f_{y}=\sigma_{\text{1}}-\sigma_{\text{3}}M_{\phi }+\text{2}c\sqrt{M_{\phi }}$$ (3)

$$f_{j}=\sigma_{\text{3}}-\sigma^{t}$$ (4)

where *σ*_1_, *σ*_3_ respectively is the maximum and minimum principal stresses, *c* and *φ* respectively is the cohesion and internal friction angle, *σ*^*t*^ is the tensile strength and have


$$M_{\varphi }=\frac{\text{1}+\text{sin }\varphi }{\text{1}-\text{sin }\varphi }$$ (5)

Different parameters are used according to different calculation models: in the Mohr-Coulomb model it requires the input of BULK (bulk modulus K), SHEAR (shear modulus G), COHESION (viscous force), FRICTION (friction angle), and TENSION (tensile strength) of the material; in the fully elastic model it requires the input of BULK (bulk modulus K), SHEAR (shear modulus G) parameters, etc.

### 4.3. Selection of calculation parameters

The fill material was taken from the residual weathered soil on the nearby hillside. The shallow part of the dam surface contains sand pebbles and crushed stones, with a small amount of weathered fragments locally, and the overall soil quality of the dam material is relatively uniform. In the numerical calculation, according to the engineering geological survey report of the project and relevant hydrological data, and with reference to the Code for Design of Embankment Dam Safety Reinforcement and the Manual of Rock Mechanics Parameters [19,20], the values of the mechanical parameters of the calculation model are shown in Table 1.

**Table 1:** Physical and mechanical parameters of the dam body.

Partition Calculation parameters	Bulk modulus (MPa)	Cohesion (kPa)	Internal friction angle (*°*)	Gravity (kN/m³)	Shear modulus (MPa)	Shear expansion angle (*°*)
Grouting curtain zone	42	47	42	27	14	12
Non-grouting curtain zone	37	32	32	22	9	10

## 5. Results

The middle section (0 + 100) of earth dam is selected as the typical section, and the finite difference software FLAC3D is used to carry out the simulation calculation, the analysis results are as follows.

### 5.1. Plastic zone distribution

**Fig 5:**
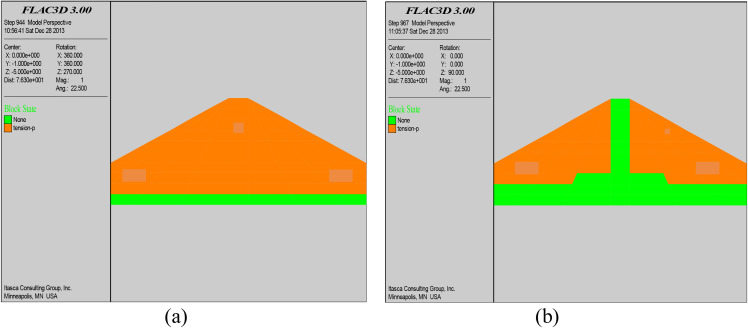
Plastic zone before grouting (a); Plastic zone after grouting (b).

Fig 5 shows the distribution of the plastic zone before and after grouting in the middle section (0 + 100 section) of the dam. From the figure, it can be seen that due to the poor construction quality of the dam, a large plastic zone appears inside the dam body before grouting, and tensile stresses appeared in some parts. After grouting, the internal stress of the dam body is adjusted due to the reinforcement effect of cement slurry on the seepage and compaction between the fill of the dam body, and a complete impervious curtain is formed near the axis of the dam body. The area of plastic zone after grouting decreased 23% compared with that before grouting, and the effect of grouting reinforcement is obvious, and the stability of the dam body is greatly enhanced.

### 5.2. Stress analysis of the dam body

**Fig 6:**
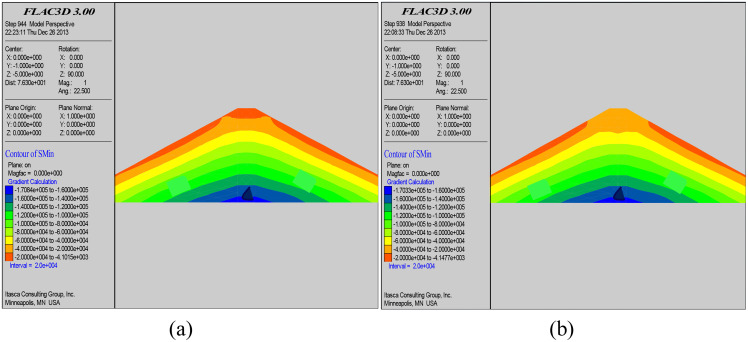
Contour map of minimum principal stress before grouting (a); Contour map of minimum principal stress after grouting (b).

**Fig 7:**
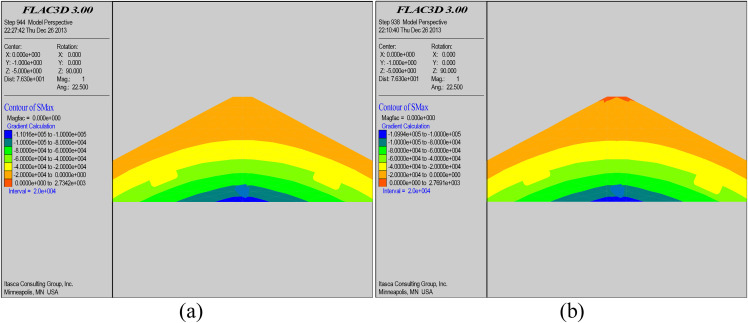
Contour map of maximum principal stress before grouting (a); Contour map of maximum principal stress after grouting (b).

Figs 6 and 7 respectively are the contour maps of maximum and minimum principal stresses before and after grouting and reinforcement of the dam body. From the figures, it can be seen that the distribution of both maximum and minimum principal stresses in the dam body has changed after the grouting and seepage control reinforcement treatment. A complete grouting curtain zone was formed inside the dam body after the grouting and reinforcement treatment, and the dam body stress was adjusted twice. Although the maximum and minimum principal stresses are still symmetrically distributed along the dam axis, the effective stresses of both maximum and minimum principal stresses inside the grouting curtain zone have been reduced. While the total stresses in the area far away from the grout curtain zone are basically unchanged. The reason for the variation of the maximum and minimum principal stresses is mainly due to the wet collapse and consolidation of the dam body caused by the precipitation of the slurry during the grouting action, and the mutual extrusion and coupling of the slurry and the dam body.

**Fig 8:**
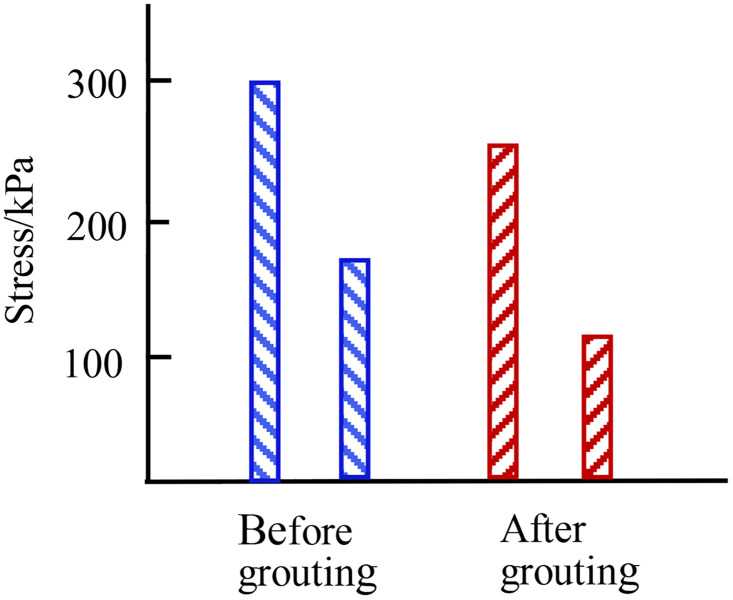
Bar chart of maximum and minimum principal stress changes.

The changes in the maximum and minimum principal stresses of the dam before and after grouting were plotted as bar charts, as shown in Fig 8.The maximum stress is reduced by 16%.

### 5.3. Dam displacement analysis

**Fig 9:**
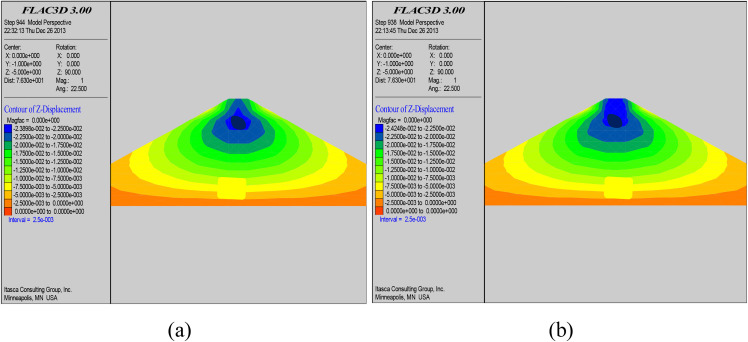
Vertical displacement cloud before grouting (a); Vertical displacement cloud after grouting (b).

Fig 9 is the vertical displacement cloud diagram of the dam body before and after grouting at section 0 + 100. From the figure, it can be seen that the vertical displacement of the dam body changed before and after grouting. The maximum vertical displacement before grouting is 22.4 cm, while the maximum vertical displacement after grouting is 24.2 cm, which is increased than before grouting. This is due to the precipitation of the slurry during grouting that caused wet collapse and consolidation of the dam body, increased water content of the soil on both sides of the slurry joints, and increased relative density of the soil body. After grouting, the deformation is stable and the dam soil is more compacted.

YG73 piezoelectric stress gauge and TCRP total station were used on site to monitor the stress state, vertical and horizontal displacement of the dam body. Through the analysis of monitoring data, it was found that the FLAC3D simulation results in this article are basically consistent with the on-site monitoring results.

## 6. Conclusion

In this paper, the action mechanisms of penetration grouting, compression grouting and splitting grouting are described, and the stability of seepage control and reinforcement of a diseased reservoir in Guangxi Province is analyzed and calculated by using FLAC3D finite difference program.

(1) With the continuous injection of grout and the increasing grouting pressure, criss-crossing network of grout veins is generated near the grouting borehole. The grout veins squeeze the soil to form a grout vein skeleton. The normal stress of the soil layer and the strength and stiffness of the soil are significantly increased.
(2) After the grouting reinforcement is completed, the plastic zone in the dam body decreased 23% and the stability of the dam body is obviously enhanced; a complete grouting curtain zone is formed inside the dam body and the stress of the dam body is reduced by 16%; the deformation of the dam body is stable, the soil of the dam body is more compact and the grouting effect is obvious.
(3) Due to the concealment of the grouting process and the complexity of the grouting mechanism, the mechanism issues such as the boundaries and distribution range of the modes of action of slurry infiltration, compacting or splitting need further in-depth study; and the constitutive model of grouting curtain zone and non-grouting curtain zone may not be fully consistent with the actual situation, and more accurate numerical calculation requires secondary development of the constitutive model embedded in FLAC3D.
